# Tramways and Healthy Dwellings

**Published:** 1897-01-02

**Authors:** 


### Tramways and Healthy Dwellings.

Those who are responsible for the civic administration of London have before them knotty problems—the question of tramways and the question of workmen's dwellings—which are intimately related the one to the other. Both of them are health problems, and they are linked together in this wise, that the necessity for municipal, as opposed to or supplementary to commercial action in regard to workmen's dwellings arises from the fact that it is the action of the sanitary authorities in clearing unhealthy areas that has necessitated the endeavour to provide healthy dwellings in place of those which have been destroyed, and that this necessity hinges on the answer to that other question, whether the municipality, by the proper use of its thoroughfares, could enable those workmen who wish to live in more salubrious neighbourhoods to get to them with reasonable speed and with reasonable cheapness. If the County Council can provide healthy dwellings for the workers of the metropolis near their work they have no special sanitary obligation laid upon them to facilitate the means of transit into the suburbs; but if, as is the fact, they cannot do so, then the obligation laid upon them to increase the means of transit is great. On the other hand, if by the full and proper use of their thoroughfares a large proportion of the workers can be carried out into open and healthy suburbs, the authorities are to that extent relieved of the obligation to enter upon costly and more or less ineffectual attempts at block building in densely-populated districts. Looking at the matter, then, from a health point of view, one cannot but feel that a great deal of the discussion that has recently taken place in regard to the tramways has been beside the mark. The streets belong to the public, and, right as it may be to demand the uttermost farthing of rent, if it is right at all to let out the streets for money-making purposes, a far more important thing from a public point of view is to develop to the utmost their carrying power. To the sanitarian, who recognises how utterly ineffectual have been all the attempts hitherto made to re-house the people turned out of their homes by various metropolitan improvements, it is distressing to find the discussion of the tramway question made to hinge on matters of rent instead of upon methods of development. Those who decline to travel beyond London in search of ideas may perhaps feel content to see a couple of miserable horses struggling along a slippery track in front of an over-laden tramcar, and may regard this mode of locomotion as the utmost that modern civilisation can give us. But those who have seen overhead electric traction in operation know how desperately we are behind the times in such matters. What we have to accept is the fact that in building workmen's dwellings the County Council works under the great disadvantage entailed in having to set a good example. The sanitary authorities have to control the erection of buildings by commercial companies, and it would never do for the central sanitary authority to put up buildings which in any way fell short of sanitary perfection. This means expense and consequent high rents, and in the end it means that instead of re-housing the people who have been displaced, the new buildings often only cater for a higher class of artisans, for people, in fact who, but for the attractions of the flats, would go out into the suburbs. Block building is thus defeating its own ends, and it becomes a serious question whether the health of the people would not be far better served by enforcing a far more complete development of the carrying power of the streets than by scaring away capital from the work of providing dwellings in the central parts of the metropolis. Surely the highways by which the suburbs are reached are not so æsthetically beautiful that they would be spoiled by a few trolly lines! That their carrying power would thereby be trebled seems to be generally admitted.

